# Clinical and genetic aspects of Mayer–Rokitansky–Küster–Hauser syndrome

**DOI:** 10.1007/s11825-018-0173-7

**Published:** 2018-02-21

**Authors:** Susanne Ledig, Peter Wieacker

**Affiliations:** Institute of Human Genetics, Westfälische Wilhelms-Universität, Vesaliusweg 12–14, 48149 Münster, Germany

**Keywords:** MRKH, *LHX1*, *TBX6*, *WNT9B*, MRKH, *LHX1*, *TBX6*, *WNT9B*

## Abstract

The Mayer–Rokitansky–Küster–Hauser (MRKH) syndrome [MIM 277000] is characterised by the absence of a uterus and vagina in otherwise phenotypically normal women with karyotype 46,XX. Clinically, the MRKH can be subdivided into two subtypes: an isolated or type I form can be delineated from a type II form, which is characterised by extragenital malformations. The so-called Müllerian hypoplasia, renal agenesis, cervicothoracic somite dysplasia (MURCS) association can be seen as the most severe phenotypic outcome.

The MRKH syndrome affects at least 1 in 4000 to 5000 female new-borns. Although most of the cases are sporadic, familial clustering has also been described, indicating a genetic cause of the disease. However, the mode of inheritance is autosomal-dominant inheritance with reduced penetrance. High-resolution array-CGH and MLPA analysis revealed recurrent aberrations in different chromosomal regions such as TAR susceptibility locus in 1q21.1, chromosomal regions 16p11.2, and 17q12 and 22q11.21 microduplication and -deletion regions in patients with MRKH. Sequential analysis of the genes *LHX1, TBX6* and *RBM8A*, which are located in chromosomal regions 17q12, 16p11.2 and 1q21.1, yielded in the detection of MRKH-associated mutations. In a subgroup of patients with signs of hyperandrogenaemia mutations of *WNT4* have been found to be causative. Analysis of another member of the WNT family, *WNT9B*, resulted in the detection of some causative mutations in MRKH patients.

## Zusammenfassung

Das Mayer-Rokitansky-Küster-Hauser (MRKH) Syndrom [MIM 277000] ist durch einen fehlenden Uterus und eine fehlende Vagina bei phänotypisch unauffälligen Frauen mit dem Karyotyp 46,XX gekennzeichnet. Klinisch werden beim MRKH 2 Subtypen unterteilt: die isolierte oder Typ I Form wird von der Typ II Form, bei der zusätzlich extragenitale Malformationen auftreten, unterschieden. Hierbei kann die sog. MURCS-Assoziation (MURCS: „Müllerian hypoplasia, renal agenesis, cervicothoracic somite dysplasia“) als schwerste phänotypische Ausprägung verstanden werden.

Das MRKH tritt bei ca. einem von 4000 bis 5000 weiblichen Neugeborenen auf; die meisten Fälle kommen sporadisch vor. Das Auftreten einiger familiärer Fälle weist auf eine genetische Ursache mit autosomal-dominanter Vererbung und einer reduzierten Penetranz hin.

Die Analyse von MRKH-Patientinnen mittels hochauflösender Array-CGH und MLPA führte zur Identifizierung rekurrierender Aberrationen in verschiedenen chromosomalen Regionen wie dem TAR-Suszeptibilitätslokus in 1q21.1, den Regionen 16p11.2 und 17q12 und der Mikroduplikations und –deletionsregion 22q11.21.

In den Genen *LHX1, TBX6 *und *RBM8A*, die in den chromosomalen Regionen 17q12, 16p11.2, bzw. 1q21.1 lokalisiert sind, wurden zudem MRKH-assoziierte Mutationen nachgewiesen. Hingegen werden Mutationen des Gens *WNT4* nur bei einer Subgruppe von Patientinnen mit zusätzlichen Androgenisierungserscheinungen detektiert. Interessanterweise konnten bei der Analyse eines weiteren Mitglieds der WNT-Familie, *WNT9B*, ebenfalls ursächliche Mutationen bei MRKH-Patientinnen gefunden werden.

## Clinical aspects

The Mayer–Rokitansky–Küster–Hauser (MRKH) syndrome [MIM 277000] is characterised by the congenital absence of the uterus and the upper two thirds of the vagina in 46,XX females with mostly normal ovarian function and therefore normal breast and pubic hair development. The exact description of the genital manifestation of MRKH is “uterus bipartitus solidus rudimentarius cum vagina solida” and in fact endometrium islands can be detected in a proportion of MRKH patients, leading to complications in some cases. Occasionally, the Fallopian tubes can also be affected, but the lower part of the vagina is usually unaffected. This is in good agreement with the hypothesis that the lower part of the vagina might develop from the urogenital sinus and might not be a derivative of the Müllerian ducts (MDs).

The first clinical feature is generally primary amenorrhea. Clinical examination typically reveals a normal female phenotype with breast development, axillar and pubic hair and normal external genitalia. Differential diagnosis includes isolated vaginal atresia, androgen insensitivity syndrome caused by mutations of the androgen receptor gene (*AR*) in XY individuals and *WNT4* defects characterised by MRKH and hyperandrogenism.

Diagnostics include different methods such as transabdominal ultrasound, MRI and pelviscopy.

The incidence of MRKH is about 1:4,500 newborn girls.

The MRKH syndrome can occur as an isolated or type I MRKH or in association with extragenital malformations as type II MRKH. Upper urinary tract malformations are observed in about 40%, including unilateral renal agenesis, ectopia of one or both kidneys, renal hypoplasia, horseshoe kidneys and hydronephrosis. The most frequent skeletal anomalies include malformations of the spine in 30–40% such as Klippel–Feil anomaly or scoliosis. Müllerian hypoplasia, renal agenesis, cervicothoracic somite dysplasia (MURCS) association is the most severe form of MRKH II characterised by MD aplasia, renal dysplasia and cervical somite dysplasia. Less frequently, MRKH can be associated with hearing defects including conduction defects such as stapes fixation or sensorineural deafness. Rarely, cardiac (atrial septum defect, conotruncal defects) and digital anomalies such as syndactyly, polydactyly or ectrodactyly can occur. Occasionally, associations with situs inversus, Dandy–Walker malformation, Meckel–Gruber syndrome, Bardet–Biedl syndrome, Holt–Oram syndrome or McKusick–Kaufman syndrome have been reported, leading to the assumption that—in at least some cases—MRKH can be seen as a ciliopathy.

## Embryogenetic aspects

The mammalian female and male reproductive tracts derive from the paramesonephric or MDs and mesonephric or Wolffian ducts (WDs) respectively. According to analyses of mice models, MD development includes three phases: 1) initiation, 2) cranio-caudal invagination of the coelomic epithelium into the mesonephros, and 3) elongation of the MD.

First, cells of the coelomic epithelium (Müllerian plaque) at the upper end of the urogenital ridge are specified to become MD cells. Subsequently, within the mesonephros, MD precursors invaginate into the underlying mesenchyme and migrate caudally along the length of the WDs extending posteriorly, cross the WDs until the caudal tip of the MDs reaches the urogenital sinus, which is of endodermal origin. It was suggested that the cells forming the MDs were of WD origin, but Orvis and Behringer were able to show that the elongation of the MDs is accomplished predominantly by a small group of cells proliferating at the tip of the MDs. These cells are tightly associated with the WDs and are guided by them [31]. The first phase of MD development (specification and invagination) occurs independently from the WDs, but in the next phase of elongation it is dependent on the presence of the WDs and furthermore on the expression of an elongation signal (Wnt9b, wingless-type MMTV integration site family, member 9b).

Although the MDs and WDs are of different origin, they coexist during embryogenesis in both sexes until genetic sex triggers the differentiation of the indifferent gonad into ovary or testis respectively. In females, the MDs give rise by fusion to the utero-vaginal duct, which differentiates into the uterus and the upper part of the vagina, whereas the unfused part of the MDs develops into the Fallopian tubes. In males, the testicular Sertoli cells secrete a glycoprotein, the anti-Müllerian hormone (AMH), which causes the regression of the MD. Targeted mutagenesis in the mouse has identified several genes that are essential for proper development and differentiation of the female reproductive tract.

The mice genes required for female reproductive tract development include paired-box-gene 2 (*Pax2*), LIM homeobox 1 (*Lhx1*)*, *wingless-type MMTV integration site family, member 4 (*Wnt4*), wingless-type MMTV integration site family, member 7a (*Wnt7a*), empty spiracles homeobox 2 (*Emx2*), hepatocyte nuclear factor 1-beta (*Hnf1b*)*, *wingless-type MMTV integration site family, member 5a (*Wnt5a*), dachshund homolog 1 (*Dach1*)*, *dachshund homolog 2 (*Dach2*), wingless-type MMTV integration site family, member 9b (*Wnt9b*), and genes of the abdominal *B Hoxa* cluster.

The homeodomain transcription factor encoding gene *Pax2* is expressed in the developing kidney and in the epithelium of the MD and the WD. According to its expression mice deficient in *Pax2* lack kidneys and genital ducts in both sexes [18]. MD development is initiated by the expression of *Pax2 *together with homeodomain coding gene *Lhx1* in the coelomic epithelial cells and specifies them for a Müllerian fate. Furthermore, *Pax2* is also essential for the next steps in MD development, the elongation and maintenance of the MD. Therefore, in *Pax2*-deficient mice the anterior portion of the MD initially forms, but then degenerates, while the urogenital sinus still gives rise to the bladder and urethra [18]. *Wnt4* and *Lhx1* are expressed in the embryonic mesonephric mesenchyme surrounding the newly formed MD and both are required for embryonic MD development. In *Wnt4*^-/-^ female mice, the absence of MD formation and in contrast stabilisation of WD suggest an essential role of Wnt4 in repressing male development in the XX gonad [45]. Furthermore, Jeays-Ward et al. showed that the masculinised phenotype of Wnt4^-/-^ female mice originates because of the disturbance of endothelial and steroidogenic cell migration into the developing XX gonad, provoking the formation of a male-specific coelomic blood vessel and production of steroids in the female gonad [16]. Possibly, *Wnt4* is acting downstream of *Lhx1* and *Wnt9b* and initiates the MD invagination [25, 45]. Wnt4 induces expression of Wnt7a [45]. Female mice lacking *Wnt7a* are infertile owing to abnormal differentiation of the uterus and oviduct [33]. The LIM domain-expressing gene,* Lhx1* is essential for the development of the epithelial cells of MDs and WDs. Therefore, female *Lhx1* knockout mice lack the uterus and the upper part of the vagina, whereas male mice are deficient in the WD derivatives [18].

*Emx2 *is another homeodomain transcription factor coding gene that is expressed in the epithelial cells of the urogenital tract. Consequently, *Emx2-*mutant mice lack kidneys, ureters, gonads and genital tracts and die soon after birth [26]. However, in these mice, WDs initial develop, but then degenerate. The POU domain-containing *Hnf1ß* is essential for general epithelial differentiation and is expressed in very early urogenital tract formation, continuing into adulthood [13]. Wnt5a is expressed in mesenchymal cells of the uterus, cervix and vagina and is required for the growth of the female reproductive tract [25]. Therefore, in mice deficient in *Wnt5a*, the cervix and the whole vagina are absent [25]. Female mice double mutant for the putative transcriptional cofactors *Dach1/Dach2* show a severe disruption of MD development [14]. The adequate development, fusion and resorption of the separating wall between the MDs seem to be induced by the WDs [1]. Thus, it is known, that the WD secretes Wnt9b, which serves as a canonical Wnt signal essential for caudal MD extension [9]. In *Wnt9b* -deficient mice, the MDs start to invaginate, but there is no elongation caudally. Interestingly, the WDs are unaffected [9]. Different genes of the Hox family play a major role in body patterning and organogenesis and are expressed during the development of the female genital tract in different areas of the MD. The expression of the different Hox genes divides the homogeneous MD into segments along the anterior–posterior axis with each segment developing into different structures according to their 3’–5’order in the *Hox* cluster: *Hoxa9* is expressed in the subsequent oviduct, *Hoxa10* in the developing uterus, *Hoxa11* in the progenitor of the lower uterine segment and cervix and *Hoxa13* in the cervix and upper vagina [47]. Mutations of either *Hoxa10* or *Hoxa11* result in uterine factor infertility in mice due to an implantation defect in the uterus [39].

## Genetic aspects

Currently, the genetics of MRKH remains elusive. There is a risk of recurrence in relatives, but most cases of MRKH are sporadic. Familial cases can be explained by autosomal dominant inheritance with reduced penetrance and variable manifestation. However, oligogenic or polygenic inheritance has also been discussed [20].

There are some reports of monozygous twins discordant for MRKH, which may be explained by mosaicism or imprinting effects. Recently, insights into genetics and the pathogenesis of MRKH have come from genetic techniques such as array CGH.

Until recently, *WNT4 *deficiency was the only known monogenetic cause for MRKH, but different groups identified by multiplex ligation-dependent probe amplification (MLPA) and genome-wide array comparative genomic hybridisation (CGH) microimbalances affecting new genes that play a role in the pathogenesis of this condition. So far, different recurrently affected chromosomal regions have been identified with the following frequencies: ~1% in 1q21.1, ~1% in 16p11.2, ~6% in 17q12 and ~4% in 22q11.21 [20, 27]. Table 1 summarises the phenotypes of MRKH patients with imbalances in the above-mentioned recurrently affected regions.

**Table 1:** Phenotypes of MRKH patients with imbalances in recurrently affected regions 1q21.1, 16q11.2, 17q12 and 22q11.21

Locus	Copy number/size	Causative genes(s)	Phenotype	Reference
1q21.1	Dup/2.7 Mb	*RBM8A*	Complete uterine + vaginal agenesis, fused external labia, ovaries undetectable; inherited by unaffected mother	Cheroki et al. (2008; [11])
1q21.1	Del/0.378 Mb	*RBM8A*	Müllerian aplasia type II, TAR syndrome	Ledig et al. (2011; [20])
16q11.2	Del/0.55 Mb	*TBX6*	MURCS, hypoplasia of the wrist, disturbed psychomotor development, epilepsy, bilateral hearing loss	Nik-Zainal et al. (2011; [29])
16q11.2	Del/0.6 Mb	*TBX6*	Müllerian aplasia, short stature	Nik-Zainal et al. (2011; [29])
16q11.2	Del/0.55 Mb	*TBX6*	MURCS, long uterus horns, scoliosis, left atrophic kidney	Nik-Zainal et al. (2011; [29])
16q11.2	Del/0.55 Mb	*TBX6*	Müllerian aplasia	Nik-Zainal et al. (2011; [29])
16q11.2	Del/0.53 Mb	*TBX6*	Müllerian aplasia	Sandbacka et al. (2013; [38])
16q11.2	Del/0.53 Mb	*TBX6*	Müllerian aplasia	Sandbacka et al. (2013; [38])
16q11.2	Del/0.53 Mb	*TBX6*	Müllerian aplasia	Sandbacka et al. (2013; [38])
16q11.2	Del/0.53 Mb	*TBX6*	Müllerian aplasia	Sandbacka et al. (2013; [38])
16q11.2	Del/0.53 Mb	*TBX6*	Müllerian aplasia	Sandbacka et al. (2013; [38])
17q12	Del/1.2 Mb	*LHX1, HNF1B*	Müllerian aplasia, mild dysmorphic features, onychodystrophy, mental impairment, seizures	Cheroki et al. (2008; [11])
17q12	Del/1.5 Mb	*LHX1, HNF1B*	Müllerian aplasia, mild dysmorphic features	Bernardini et al. (2009; [3])
17q12	Del/1.5 Mb	*LHX1, HNF1B*	Müllerian malformations with right unicornuate uterus, no cavitating rudimentary left horn, right haematosalpinx and surgically corrected agenesis of the upper and middle thirds of the vagina, bilaterally multicystic kidneys	Bernardini et al. (2009; [3])
17q12	Del/1.8 Mb	*LHX1, HNF1B*	Müllerian aplasia	Ledig et al. (2011; [20])
17q12	Del/1.4 Mb	*LHX1, HNF1B*	Müllerian aplasia, unilateral kidney agenesis	Ledig et al. (2011; [20])
17q12	Del/1.4 Mb	*LHX1, HNF1B*	Müllerian aplasia	Nik-Zainal et al. (2011; [29])
17q12	Del/1.4 Mb	*LHX1, HNF1B*	MURCS, left kidney agenesis with absent ureter, pelvic misalignment, type II diabetes	Nik-Zainal et al. (2011; [29])
17q12	Del/1.4 Mb	*LHX1, HNF1B*	MURCS, absent right kidney, left pelvic kidney, right convex kyphoscoliosis	Nik-Zainal et al. (2011; [29])
17q12	Del/1.4 Mb	*LHX1, HNF1B*	MURCS, mild scoliosis	Nik-Zainal et al. (2011; [29])
17q12	Del/1.7 Mb	*LHX1, HNF1B*	Müllerian aplasia	Sandbacka et al. (2013; [38])
22q11.21	Del/2.6 Mb	?	Müllerian malformation with vaginal agenesis and rudimentary uterus, right kidney agenesis, scoliosis, mild to moderate learning disabilities, mild dysmorphic features	Cheroki et al. (2006; [10]); Cheroki et al. (2008; [11])
22q11.21	Del/0.39 Mb	?	Müllerian aplasia	Ledig et al. (2011; [20])
22q11.2	Del/0.39 Mb	?	MURCS, 1‑cm blind ending vagina, rudimentary uterus, normal right ovary, streak left ovary, fused pelvic kidney, fused vertebrae, scoliosis, hypoplastic first ribs, flattened sacrum, dysplastic auricles, right-sided cleft lip, cleft palate, atrial septal defect, persistent left superior vena cava, unroofed coronary sinus, patent ductus arteriosus, multiple nevi, left hypoplastic thumb, hypoplastic middle phalanx, absent distal phalanx second digit, absent middle and distal phalanges, fifth digit	Nik-Zainal et al. (2011; [29])
22q11.21–q11.23	Dup/3.5 Mb	?	Müllerian aplasia	Ledig et al. (2011; [20])

*TAR* thrombocytopaenia/absent radius, *MRKH* Mayer–Rokitansky–Küster–Hauser syndrome,* MURCS* Müllerian hypoplasia, renal agenesis, cervicothoracic somite dysplasia

### 1q21.1

Imbalances in 1q21.1 affecting the so-called common thrombocytopaenia/absent radius (TAR; MIM27400) susceptibility locus have been identified in patients with or without signs of the TAR syndrome (hypomegakaryocytic thrombocytopaenia, bilateral absence of the radius in the presence of both thumbs) in addition to Müllerian malformations (Table 1; [11, 20]). In an MRKH type II patient with signs of TAR syndrome a deletion affecting the TAR susceptibility locus has been identified [20]. In a second patient, a gross duplication of approximately 2.7 Mb, also overlapping the common TAR deletion interval has been described [11]. Most TAR patients carry deletions of different sizes, but always affecting a 200-kb gross common deletion interval, the TAR susceptibility locus. Rarely, malformations of the genitourinary anomalies have been observed in patients with TAR syndrome including horseshoe kidney, hypoplasia of the uterus and vagina, and renal pelvis dilatation. Furthermore, it is known from analysis of TAR patients that about 75% have inherited the deletion from an unaffected parent [17]. Therefore, the authors supposed that in addition to the rare deletion, a second frequent change, possibly a frequent variant, is needed for the phenotypic manifestation of TAR. However, mutational analysis of 10 genes, located in the minimal deletion interval, in 3 patients revealed in a first approach no second causative mutation [17]. However, recently in all patients analysed, one of two rare intronic regulatory polymorphisms in the *RBM8A* gene, which is located in the minimal deletion interval of the TAR susceptibility locus, have been found on the second allele [2]. Furthermore, in the case of patients with clinical signs of TAR and the causative polymorphism in the regulatory region of *RBM8A*, but without the deletion in 1q21.1, nonsense mutations of *RBM8A* in a compound heterozygous manner have been found [2]. *RBM8A* encodes the Y14 protein, which is one of four core components of the exon junction complex (EJC). In *Drosophila melanogaster*, the Y14 protein is necessary for oocyte differentiation and determination of primordial germ cells [32].

All of these findings suggested a strong association between *RBM8A* and MRKH. Therefore, by performing sequence analysis of *RBM8A* in a group of 116 MRKH patients, one of the two TAR-associated variants and a second undescribed intronic variant were found with higher frequencies in the patient group in contrast to the general population [43]. Interestingly, one patient carried both *RBM8A* variants mentioned above, whereas another carried a gross duplication, which contains the Bardet–Biedl syndrome (BBS)-associated *BBS9* gene [43]. Furthermore, in a patient with MRKH I and XX gonadal dysgenesis, a heterozygous *RBM8A* missense mutation was found, making *RBM8A* an interesting candidate for MRKH syndrome associated with ovarian dysgenesis too [43].

### 16p11.2

Losses in 16p11.2 have primarily been described in combination with autism spectrum disorders, but also with epilepsy, seizures, developmental delay and learning disability, dysmorphism/congenital anomalies (abnormal head size) and obesity. Furthermore, deletions in 16p11.2 were also identified in unaffected persons. However, in an array-based study of patients with isolated and syndromic Müllerian aplasia, in 4 of the 63 patients deletions of this locus were identified, suggesting a strong association of this region with MRKH syndrome (Table 1; [29]). Among the genes deleted in the common deletion interval, *TBX6* seems to be a good candidate gene, as it encodes a conserved transcription factor, playing an essential role in developmental processes such as mesoderm formation and specification.

Sequence variants in *TBX6* are known to cause congenital scoliosis in the Chinese Han population and spondylocostal dysostosis [15]. A mouse model, the homozygous *Tbx6*^rv^ (rib-vertebrae), show a hypomorphic phenotype, with an occasionally unilateral absence of kidneys and reduced female fertility [49]. The phenotype in this mouse model and the known association between *TBX6* mutations in humans and scoliosis strongly resembles the MURCS association in humans.

Sequencing of the *TBX6* gene in two studies with MRKH patients resulted in the identification of one possible pathogenic missense and one splice site mutation in a total of four patients [38, 43]. Corresponding to the phenotype seen in mice with homozygous *Tbx6 *mutations, two of these patients also show skeletal malformations [43]. Furthermore, two known polymorphisms could be associated with Müllerian aplasia, as they were found at a higher frequency in patients in contrast to the general population [38].

### 17q12

The most recurrently affected chromosomal region in MRKH is 17q12. Different array-based studies identified deletions of 1.4–1.8 Mb in size in chromosomal region 17q12 in patients with MRKH types I and II (Table 1; [3, 11, 20, 29, 38]). Associated malformations were bilaterally multicystic kidneys, mild facial dysmorphisms [3], but also severe learning disability and seizures (Table 1; [11]). Furthermore, deletions of 17q12 can also give rise to other phenotypes without any impairment of MD.

Due to expression data and mouse models, different studies favoured *LHX1* and *HNF1B* as promising candidate genes for MRKH, but also for associated traits of the 17q12 deletion. Both genes are discussed in the following. Furthermore, the finding that the deletion size and the breakpoints observed in patients with MRKH type I are similar to those in patients with a more severe phenotype or with malformations not affecting the MD makes the involvement of other genes outside the deletion interval for this extended phenotype likely. An oligogenic mode of inheritance has also been suggested for MRKH and would explain the rare familial cases and the difficulty in identifying a single genetic cause.

#### *LHX1*

Of a total of 118 MRKH patients, we could detect in two of them heterozygosity for a frameshift mutation and a missense mutation in the *LHX1 *gene respectively [20, 21]. The frameshift causes a very early premature stop codon at amino acid position 33 and was detected in a type II patient with unilateral kidney agenesis [21]. The patient, who carries the *LHX1* missense mutation, has a type I MRKH syndrome [20]. Furthermore, three pathogenic *LHX1* mutations were found in 5 out of 112 Finnish patients with aplasia of the Müllerian ducts [38].

*LHX1* on chromosome 17 belongs to the LIM homeodomain family of transcription factors, which is implicated during embryogenesis in processes such as body axis determination, in addition to tissue and regional specification. LIM homeodomain proteins contain two tandem LIM domains followed by a central homeodomain with DNA-binding activity and a C-terminal transactivation domain, which may be involved in the transcriptional regulation of target genes. The LIM domain is a cysteine-rich double zinc finger motif that binds zinc and functions as a protein adapter module that can interact with different protein domains and regulate by this the function of different components in the transcriptional complex. Female *Lhx1*-null mice lack a uterus and oviducts together with a complete absence of both the epithelium and the mesenchyme of the female reproductive tract, while the ovaries are unaffected [18], a phenotype strongly resembling MRKH syndrome in humans. Additionally, mice lacking *Lhx1* lack kidneys and are anencephalic [40]. Most embryos deficient of *Lhx1* die at embryonic day E10 because of defects in allantois differentiation, and only a few are stillborn.

Expression of *Lhx1* in the epithelium of the developing MDs in a mouse model is dynamic with onset of the expression at embryonic day 11.5 (E11.5) in the most anterior region of the urogenital ridge and caudal extension at E13.5 in both sexes [18]. The *Lhx1* expression in the epithelium of the MDs becomes sexually dimorphic at E14.5, with persistent strong expression until E16.5 in females and a weaker expression in males than in females consistent with the regression of the MDs in males to this point of time. Afterwards, the *Lhx1* expression also becomes downregulated in females, but persists in the differentiating oviduct. These findings correspond to an essential role of Lhx1 in the formation of the MDs in the female and suggest a further role in the development of the oviducts. Furthermore, by using a chimera assay, Kobayashi et al. showed that Lhx1 is required cell-autonomously for very early MD epithelium formation and that its expression in the Müllerian precursor cells is independent of Wnt7a, Pax2 and Wnt4 [18].* Lhx1* is also expressed in the WD in both sexes. In females,* Lhx1* expression is lost from the anterior gonadal region around E15.25, whereas *Lhx1* expression in males persists and becomes upregulated around E17.5. Interestingly, the only *Lhx1*-null male neonate lacks Wolffian derivatives, also suggesting that Lhx1 might play an essential role in the development of the male genital tract [18].

Furthermore, the specific knockout of *Lhx1* in the WD epithelium causes the lack of the WDs, but also impairs the further development of the MDs, confirming the importance of WDs for the elongation of the MDs [19].

In addition to the malformations of the MDs, *Lhx1-*minus mice lack, as mentioned above, any kidneys and fail to form normal anterior head structures [18, 40]. Renal malformations such as unilateral agenesis, ectopia of kidneys or horseshoe kidneys are quite often in MRKH, but even bilateral renal agenesis has been reported (Potter sequence). Interestingly the patient who carries the *LHX1* frameshift mutation shows unilateral renal agenesis. This phenotypic outcome is similar to observations from a conditional knockout of *Lhx1* in nephric epithelium after nephric duct development, which led to hypoplastic kidneys, hydronephrosis and unilateral agenesis [19].

*Lhx1* is expressed in the gonads and complete loss of *Lhx1* has first been described to cause defective head structures and a lack of kidneys in addition to a loss of gonads in mice [40]. However, such agonadism was not found in neonate *Lhx1*minus mice with mixed genetic background [18]. Gonadal dysgenesis and agenesis are also rarely associated with MRKH [8]. Complete gonadal dysgenesis with only fibrogenous tissue and a lack of germ cells can be distinguished from a partial form with residues of hormonal active tissues and some germ cells. A premature loss of germ cells in the female causes gonadal dysgenesis. Interestingly, mouse embryos lacking *Lhx1* activity are deficient of primordial germ cells (PGCs; [44]). Recently, it could be shown by performing conditional knockout of *Lhx1* in epiblast derivative that Lhx1 has no impact on the formation of PGCs, but influences their retention in the hindgut endoderm, resulting in the loss of PGCs subsequently [41]. These observations make *LHX1* an interesting candidate for MRKH syndrome associated with gonadal dysgenesis. However, in two previously described patients with MRKH and gonadal dysgenesis, no mutation in *LHX1* was found [20, 21], although one male patient with a 17q12 duplication has a 46,XX sex reversal [24].

LHX1 is also essential for differentiation of the central nervous system [19, 34] and there are rare reports of MRKH patients with mild mental retardation or learning disabilities [11]. Both patients with mental retardation in our collective harbour no *LHX1* mutation, but they both have a heterozygous deletion of approximately 1.4–1.8 Mb in 17q12 encompassing the *LHX1* gene [21].

#### *HNF1B*

Heterozygous mutations and whole gene deletions of the tissue-specific homeodomain transcription factor *HNF1B* gene are typically associated with renal cysts and diabetes (OMIM 137920). However, other phenotypic characteristics have also been described in association with *HNF1B* alterations. These are in good agreement with the tissues that express *HNF1B* such as kidney, pancreas, liver and the uterus. Expression of *Hnf1b* has been shown in MDs in the mouse embryo and in the inner epithelial layer in the adult mouse [13]. Therefore, in very few cases, *HNF1B* mutations have also been reported to cause, in association with renal tract malformations, abnormalities of the MDs in females [7]. However, studies analysing the *HNF1B* gene in patients with MRKH failed to identify mutations in *HNF1B* [3, 20, 21]. So far, only one case of a heterozygous *HNF1B* missense mutation and an isolated bicornuate uterus has been described [7]. Therefore, *HNF1B* mutations are associated as a cardinal feature with malformations of the renal tract and are a very rare cause of Müllerian disorders.

### 22q11.21

22q11.21 deletions are commonly associated with DiGeorge or velocardiofacial syndrome (DG/VCFS OMIM 188400/192430). DG/VCFS belongs to a group of related dysmorphic syndromes with highly variable clinical phenotypes encompassing congenital heart defects, hypocalcaemia, immunodeficiency, typical facial dysmorphism, learning, speech and behavioural disorders. Moreover, some publications showed an association between 22q11.21 deletions and Müllerian aplasia. Therefore, MRKH syndrome has been considered to be part of the spectrum of clinical features of the DG/VCFS. Deletions and duplications of the DiGeorge syndrome-associated region 22q11.21 have also been found in MRKH patients (Table 1; [11, 20, 29]). Cheroki detected a gross deletion in a patient with uterus agenesis and further features present in DG/VCFS (Table 1; [10, 11]). The deletion was disrupted by a short unaffected region containing the *TBX1* gene, which is responsible for some of the major clinical features of DGS/VCFS. The authors suggest that the non-deletion of the *TBX1* gene might be causative of the milder phenotype. A smaller deletion, also non-affecting the *TBX1* gene, has been identified in a patient with type I MRKH syndrome [20]. Furthermore, an adjacent duplication of approximately 3.4 Mb has been found in another type I MRKH patient, overlapping with the distal part of the 22q11.21 microdeletion–microduplication region.

These findings suggest that genes other than the *TBX1* gene in the 22q11.21 deletion syndrome might play a role in uterine malformations. Interestingly, analysis of MRKH patients with a commercially available DGS and DGS-like MLPA kit revealed imbalances in 22q11.2 and in the DGS-like phenotype associated regions 4q34-qter, 8p23.1 and 10p14 [28].

In addition to recurrent aberrations, array CGH-based studies also identified various interesting private losses and gains such as 2p24.1–24.3, 7p14.3 and Xq21.31 [11, 20, 29].

### Members of the WNT family

#### *WNT4*

*WNT4*, which maps to human chromosome 1, is a member of the WNT family of structurally related and highly conserved genes that encode secreted signalling factors that regulate a broad range of developmental processes, but has also been implicated in carcinogenesis. WNT4 is known to be essential for the development of the female reproductive tract, whereas it has been shown to play in the female gonad a double role, on the one hand by controlling the female development and on the other hand by preventing testes formation.

Heterozygous mutations in the *WNT4* gene have been associated with MRKH in humans [4, 5, 35, 36]. The 4 patients described so far displayed an agenesis or hypoplasia of the Müllerian derivatives, but also clinical or biochemical signs of hyperandrogenism (hirsutism, acne, elevated plasma testosterone levels). These findings are in good agreement with the phenotype found in *Wnt4*-deficient mice, which fail to develop MD and are masculinised. However, unilateral renal agenesis has been identified in females with heterozygous *WNT4* mutations. Functional analysis of the *WNT4* mutations revealed failure of post-transcriptional lipid modification, misfolding and formation of intractable aggregates, defects in receptor-binding and partial deregulation of enzymes involved in ovarian androgen biosynthesis [6, 36].

Studies involving classical MRKH patients failed to identify *WNT4* mutations [10, 12]. Therefore, it has been suggested that MRKH syndrome with signs of androgenisation due to heterozygous *WNT4 *mutations is a distinct clinical entity that can be delineated from typical or classic MRKH. Moreover, because of its role in gonadal development, folliculogenesis can also be disturbed in affected women [35].

#### *WNT9B*

However, alterations in other members of the complex WNT signalling pathway have been suggested as being causative, but no mutation in *WNT5A, WNT7A* and *WNT9B* could be detected in 11 MRKH patients [37].

Despite these findings, *WNT9B* seemed to be a good candidate, as *Wnt9b*^*-/-*^ female mice have no uterus and upper part of the vagina, but have normal ovaries, which is comparable to the MRKH phenotype in women [9]. Furthermore, Carroll et al. showed in the same work that Wnt9b acts upstream of Wnt4 in the development of the urogenital tract and is essential for the development of mesonephric and metanephric tubules and caudal extension of the Müllerian ducts in mice.

A first association between *WNT9B* and MRKH was found in a Chinese study with 42 patients, in which two possible pathogenic *WNT9B* mutations were detected in one patient [46]. Although it was unknown if these two mutations had been in cis or trans, the authors suggested a synergistic effect. In contrast, another Chinese study found no association between anomalies of the Müllerian ducts and mutations in *WNT9B* [42].

However, by analysing *WNT9B* in a group consisting of 59 MRKH and 50 MRKH II patients, Waschk et al. identified in five of the MRKH I patients potential pathogenic mutations (one nonsense and four missense mutations) [48], but no *WNT9B* mutation was detected in MRKH II patients. Interestingly, previous studies showed that two of the patients with a *WNT9B* mutation carried either an additional deletion of *LHX1* or a missense mutation in *TBX6* [20, 43], suggesting digenic inheritance in MRKH. Interestingly, it was shown that the expression of Wnt9b in *Lhx1*-deficient mice is markedly altered [34]. All of these findings suggest a common pathway in MRKH syndrome with WNT9B acting upstream of WNT4 and LHX1.

Furthermore, in the past, the possible involvement of other genes in the pathogenesis of MRKH has been tested.

Mutations affecting *AMH*, which initiates regression of MDs during male embryonal development, anti-Müllerian hormone receptor (*AMHR*) and various homeobox (HOX) genes, have been excluded as causative factors for MRKH syndrome [30]. Furthermore, mutational analysis of *HOXA10* and *HOXA11* in a small group of patients with malformations of the female genital tract revealed only one missense variant of unknown pathogenicity, which was also present in the patients’ unaffected mother [23].

Finally, it should be considered that exogenous factors such as diethylstilbestrol (DES), which functions as a strong oestrogen, may be involved in the pathogenesis of MRKH syndrome.

## Conclusion

Mayer–Rokitansky–Küster–Hauser syndrome is a phenotypically and genetically very heterogeneous disorder and has an incidence of 1:4,500 newborn females. Most of the cases are sporadic, but analyses of the few reported familial cases suggest an autosomal-dominant inheritance with reduced penetrance. As array CGH analyses in MRKH patients identified recurrent aberrations in chromosomal regions 1q21.1, 16p11.2, 17q12 and 22q11.21 respectively, array CGH analyses should be performed in women with the suspected diagnosis of MRKH syndrome. These recurrent aberrations are associated with highly variable clinical phenotypes and can also cause further disorders, e. g. in the case of 22q11.21 deletion heart defects or 17q12 deletion maturity-onset diabetes of the young (MODY) due to deletions of *HNF1B*.

Furthermore, the clinical overlap of MRKH syndrome with different ciliopathies suggests that MRKH syndrome might also be a ciliopathy.

By analysing candidate genes, being located in these aberrations, mutations in genes such as *LHX1, RBM8A* and *TBX6* have been identified as being causative of MRKH syndrome. However, in *WNT4*, which is associated with a distinct clinical entity of MRKH syndrome and signs of hyperandrogenism, and in its family member *WNT9B, *causative mutations have also been detected. Furthermore, in some patients with fusion anomalies of the MDs such as uterus didelphis, the same causes as for MRKH syndrome, e.g. deletions in 17q12, duplications of chromosomal region 22q11.21, and variants in *WNT9B, TBX6* and *RBM8A* have been described, suggesting that Müllerian fusion anomalies and MRKH syndrome might have a partially common aetiology [22, 43, 48].

New insights can be expected from studies on large cohorts of well-characterised patients in combination with technologies such as next-generation sequencing.

## Aspects of genetic counselling

- MRKH syndrome is a rare, heterogeneous disease characterised by the absence of a uterus and the upper two thirds of the vagina in 46,XX females.
- Most cases are sporadic, but familial occurrence is well documented and indicates autosomal-dominant inheritance with variable manifestation.
- MRKH syndrome is frequently associated with malformations, especially of the kidneys (unilateral renal agenesis 30%), skeleton (10–15%), cardiac anomalies (2–3%) and deafness (2–3%).
- Siblings of MRKH patients can also show, for example, malformations of the MDs or associated anomalies.
- In about 10%, causative microdeletions and microduplications can be detected by array CGH.
- The detection of a microdeletion in chromosomal region 16p11.2, 17q12 or 22q11.2 in a MRKH patient can also have implications for other family members. Notably, those imbalances can be inherited by unaffected parents and can be associated with autism (16p11.2), MODY (17q12) or cardiac malformations (22q11.21), which can also occur in male family members.
- An increasing number of genes responsible for MRKH syndrome such as *LHX1, TBX6, WNT9B*, and *WNT4* have been identified.
- In fusion anomalies of the uterus, the same causes of MRKH syndrome can be identified. Both MRKH syndrome and fusion anomalies of the uterus can be observed in the same family.
- Genetic diagnosis includes array CGH and next-generation sequencing.
- Various surgical procedures are available for building a neovagina. In a few cases, uterus transplantation has been performed to enable pregnancy.
